# Elastin-specific MRI of extracellular matrix-remodelling following hepatic radiofrequency-ablation in a VX2 liver tumor model

**DOI:** 10.1038/s41598-021-86417-6

**Published:** 2021-03-25

**Authors:** Federico Collettini, Carolin Reimann, Julia Brangsch, Julius Chapiro, Lynn Jeanette Savic, David C. Onthank, Simon P. Robinson, Uwe Karst, Rebecca Buchholz, Sarah Keller, Bernd Hamm, S. Nahum Goldberg, Marcus R. Makowski

**Affiliations:** 1 Department of Radiology, Charité – Universitätsmedizin Berlin, Corporate Member of Freie Universität Berlin, Humboldt-Universität Zu Berlin, and Berlin Institute of Health, Charitéplatz 1, 10117 Berlin, Germany; 2 Department of Veterinary Medicine, Institute of Animal Welfare, Animal Behavior and Laboratory Animal Science, Freie Universität Berlin, Königsweg 67, 14163 Berlin, Germany; 3 Department of Radiology and Biomedical Imaging, Yale University School of Medicine, 333 Cedar Street, New Haven, CT 06520 USA; 4 Lantheus Medical Imaging, North Billerica, MA USA; 5 Institute of Inorganic and Analytical Chemistry, Westfälische Wilhelms-Universität Münster, Münster, Germany; 6 Department of Radiology, Hadassah Hebrew University Medical Center, 9112001 Jerusalem, Israel; 7 School of Biomedical Engineering and Imaging Sciences, King’s College London, St Thomas’ Hospital, London, SE1 7EH UK; 8 BHF Centre of Excellence, King’s College London, London, UK; 9 Berlin Institute of Health (BIH), Anna-Louisa-Karsch 2, 10178 Berlin, Germany; 10 Department of Radiology, TU München, Ismaninger Straße 22, 81675 München, Germany

**Keywords:** Molecular medicine, Preclinical research, Translational research

## Abstract

Hepatic radiofrequency ablation (RFA) induces a drastic alteration of the biomechanical environment in the peritumoral liver tissue. The resulting increase in matrix stiffness has been shown to significantly influence carcinogenesis and cancer progression after focal RF ablation. To investigate the potential of an elastin-specific MR agent (ESMA) for the assessment of extracellular matrix (ECM) remodeling in the periablational rim following RFA in a VX2 rabbit liver tumor-model, twelve New-Zealand-White-rabbits were implanted in the left liver lobe with VX2 tumor chunks from donor animals. RFA of tumors was performed using a perfused RF needle-applicator with a mean tip temperature of 70 °C. Animals were randomized into four groups for MR imaging and scanned at four different time points following RFA (week 0 [baseline], week 1, week 2 and week 3 after RFA), followed by sacrifice and histopathological analysis. ESMA-enhanced MR imaging was used to assess ECM remodeling. Gadobutrol was used as a third-space control agent. Molecular MR imaging using an elastin-specific probe demonstrated a progressive increase in contrast-to-noise ratio (CNR) (week 3: ESMA: 28.1 ± 6.0; gadobutrol: 3.5 ± 2.0), enabling non-invasive imaging of the peritumoral zone with high spatial-resolution, and accurate assessment of elastin deposition in the periablational rim. In vivo CNR correlated with ex vivo histomorphometry (ElasticaVanGiesson-stain, y = 1.2x − 1.8, R^2^ = 0.89, p < 0.05) and gadolinium concentrations at inductively coupled mass spectroscopy (ICP-MS, y = 0.04x + 1.2, R^2^ = 0.95, p < 0.05). Laser-ICP-MS confirmed colocalization of elastin-specific probe with elastic fibers. Following thermal ablation, molecular imaging using an elastin-specific MR probe is feasible and provides a quantifiable biomarker for the assessment of the ablation-induced remodeling of the ECM in the periablational rim.

## Introduction

Radiofrequency ablation (RFA) represents a well-established and guideline-approved minimally-invasive therapy for patients with very early and early stage hepatocellular carcinoma (HCC)^1–3^. The efficacy of this potentially curative technique has been validated in randomized controlled clinical trials reporting convincing results in selected patients^4,5^. In recent years, several studies have challenged the sufficiency of merely local considerations for focal ablation by demonstrating that focal tumor destruction may have widespread effects extending far beyond the zone of ablation. In this regard, there is ever increasing experimental evidence suggesting that focal thermal ablation may provoke a host of secondary tissue reactions resulting in both anti-oncogenic (e.g. abscopal) as well as pro-oncogenic effects^4^. While the former have been related to the stimulation of a protective immune response induced by thermal ablation, the latter have been shown to be linked to the inflammatory reaction and the wound healing process secondary to focal tumor destruction^6–11^. The wound healing process, implemented by liver tissue in response to RFA, comprises local tissue reactions such as inflammatory cell infiltration and extracellular matrix (ECM) remodeling in the periablational rim as well as global alterations such as increased expression of inflammatory cytokines and growth factors^6–9,12–15^.

RFA-induced ECM remodeling in the periablational rim is characterized histologically by the progressive formation of structural proteins, in particular collagen and elastin, resulting in increased matrix stiffness with dramatic changes in the mechanical properties of the tumor microenvironment (TME)^16–19^. In recent years, several groups have demonstrated, that RFA-induced excessive ECM deposition significantly promotes proliferation, motility and progression of heat-exposed residual HCC cells through stiffness-dependent upregulation of the extracellular signal-regulated kinase (ERK) signaling cascade and more importantly that matrix stiffness-induced ERK activation and in vivo tumor progression can be inhibited by the administration of adjuvant therapies such as vitamin K1 and sorafenib^16–19^. As ECM remodeling cannot be depicted with conventional imaging methods, a non-invasive molecular imaging tool able to visualize and quantify RFA-induced ECM remodeling in vivo would allow to noninvasively monitor and possibly counteract excessive ECM deposition following hepatic RFA^20,21^. ECM remodeling in general and elastin deposition in particular has been previously imaged in vivo using the gadolinium‐based elastin‐specific magnetic resonance contrast agent (ESMA) in the setting of different cardiovascular and nephrological diseases^22–24^.

The purpose of our study was to translate the previously established techniques to the oncologic scenario and evaluate the use of ESMA for the non-invasive monitoring and quantification of ECM remodeling in the periablational rim following focal RFA in a VX2 rabbit liver tumor model.

## Results

**Figure 1:**
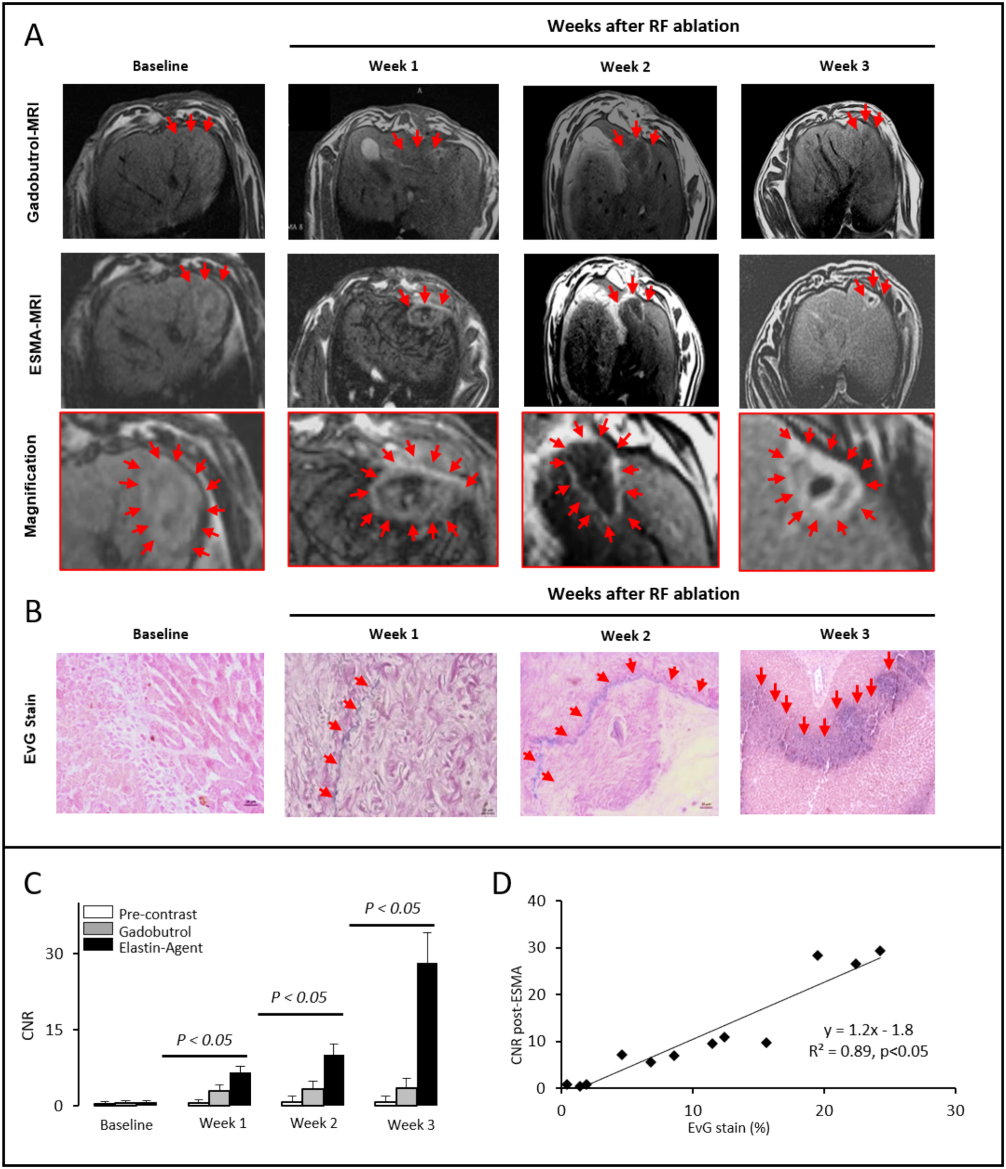
Elastin-specific MR imaging enables monitoring of ECM remodeling in the periablational rim following hepatic RFA. (**A**) Representative axial T1-weighted MR images acquired at four different time points following RF ablation (week 0/baseline, week 1, week 2 and week 3). On gadobutrol-enhanced T1-weighted images (first row) ablation zones appear as inhomogeneous areas of non-enhancing liver parenchyma. A thin and rim of enhancement on gadobutrol-enhanced images suggests a physiologic periablational hyperemia due to granulation tissue formation induced from thermal injury. The second a third (magnification) rows show ESMA-enhanced T1-weighted images acquired in the same animals on day 2 of each time point. While at baseline no hyperintense rim surrounding the ablation zone can be detected, ESMA-enhanced T1-weighted images acquired at weeks one, two and three after RF ablation display a steadily growing band like area of enhancing liver parenchyma surrounding the zone of ablation (white arrowheads). (**B)** Miller’s Elastica van Gieson (EvG) stained sections confirm a progressive deposition of elastic fibers around the zone of ablation starting one week after ablation and peaking three weeks after ablation. Scale bars indicate 100 µm. (**C**) Average periablational rim contrast-to-noise ratio (CNR) pre-contrast and following the administration of the contrast agents gadobutrol and ESMA. On pre-contrast scans (white bars), no significant CNR increase can be measured over time. Gadobutrol-enhanced scans (grey bars) show a minor, inflammation induced enhancement in the rim with no significant increase over time. A significant and progressive increase in CNR could be observed throughout the time points in the periablational rim using the elastin-specific agent (black bars), indicating a strong binding of the elastin-specific MR-probe in the periablational rim. (**D**) In the scatter-plot, the in vivo measured CNR showed a strong correlation (p ≤ 0.05) with the ex vivo Elastica-van-Giesson-staining.

RFA was technically successful in all animals. The mean diameter of the ablation zone was 10.4 mm. The difference in the diameter of the ablation zone in the four groups was not statistically significant (p > 0.05). At ex vivo histopathologic analysis following RF ablation, no residual tumor cells could be detected in or adjacent to the zone of ablation. *EvG stain* displayed an extensive remodeling of the extracellular matrix in the liver parenchyma surrounding the ablation zone (periablational rim) characterized by a progressive up-regulation of elastin deposition. Accordingly, ESMA-enhanced T1-weighted magnetic resonance (MR) images showed a progressive enhancement of the periablational rim starting 1 week and peaking 3 weeks after ablation. In vivo and ex vivo imaging of the periablational rim over time is shown in Fig. 1.

### Ex vivo imaging of RFA-induced ECM remodeling in the periablational rim

While no significant expression of elastic fibers was observed in the periablational rim at baseline examination, sections stained for Miller’s Elastica van Gieson stain (EvG) obtained from week 1 to week 3 displayed a progressive deposition of elastic fibers surrounding the zone of ablation. At week 1 after ablation EvG stain showed a linear deposition of elastic fibers at the borders of the ablation zones forming a thin fibrotic capsule separating the coagulated liver tissue from the “healthy” surrounding liver. The thickness of the fibrotic capsule delimitating the ablation zone increased at week 2 and peaked on week 3, where the strongest elastin deposition was observed. EvG stained sections obtained 3 weeks after ablation showed a thick fibrotic cap surrounding the ablation zone. %EvG stain area displayed a steady increase over time: week 1 (%EvG stain area = 6.67; p < 0.05), week 2 (%EvG stain area = 13.17; p < 0.05) and week 3 (%EvG stain area = 22.07; p < 0.05). In the healthy liver tissue (contralateral lobe), no increased expression of elastin was observed over the time course of the study.

### In vivo molecular imaging of RFA-induced ECM remodeling in the periablational rim

ESMA-enhanced MR images showed a progressive increase in the periablational rim enhancement starting 1 week and peaking 3 weeks after ablation. Contrast-to-noise ratio (CNR) values in the rim demonstrated a gradual increase over time: week 1 (CNR = 6.57; p < 0.05), week 2 (CNR = 10.06; p < 0.05) and week 3 (CNR = 28.10; p < 0.05). In vivo CNR measurements over time using the elastic-specific probe demonstrated a tight correlation with ex vivo %EvG stain area measurements (y = 1.2x − 1.8, R^2^ = 0.89; p < 0.05). No significant enhancement of the periablational rim was measured on pre-contrast MR scans or after injection of the nonspecific control agent gadobutrol at all time points, suggesting specific binding of the elastin agent.

### Gadolinium concentration by inductively coupled mass spectroscopy (ICP-MS)

**Figure 2:**
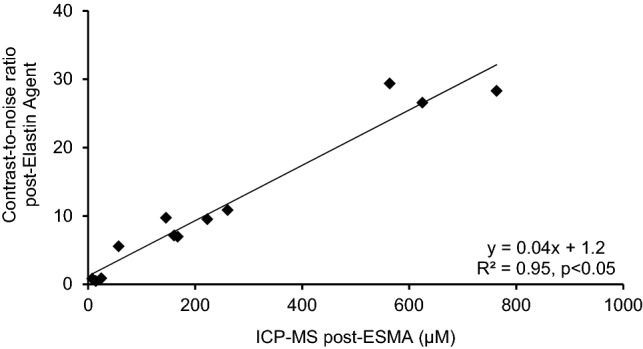
In vivo MRI signal measurements and ex vivo quantification of the elastin-specific probe in the periablational rim. Scatter plot shows a significant (p < 0.05) correlation between the contrast-to-noise ratios measured in the periablational rim and results from ICP-MS of liver tissue. This indicates a close association between the amount of in vivo binding of the elastin-specific molecular probe and the overall gadolinium detected in the ablated tissue. *ICP-MS* inductively coupled mass spectroscopy.

The average concentration of gadolinium in the periablational rim increased substantially in line with the expression of elastin throughout the different time points. The ex vivo measured gadolinium concentrations (ICP-MS) demonstrated a significant correlation with in vivo CNR (y = 0.04x + 1.2, R^2^ = 0.95, p < 0.05) (Fig. 2).

### Spatial localization of the gadolinium-based elastin-specific probe by laser coupled mass spectroscopy (LA-ICP-MS)

**Figure 3:**
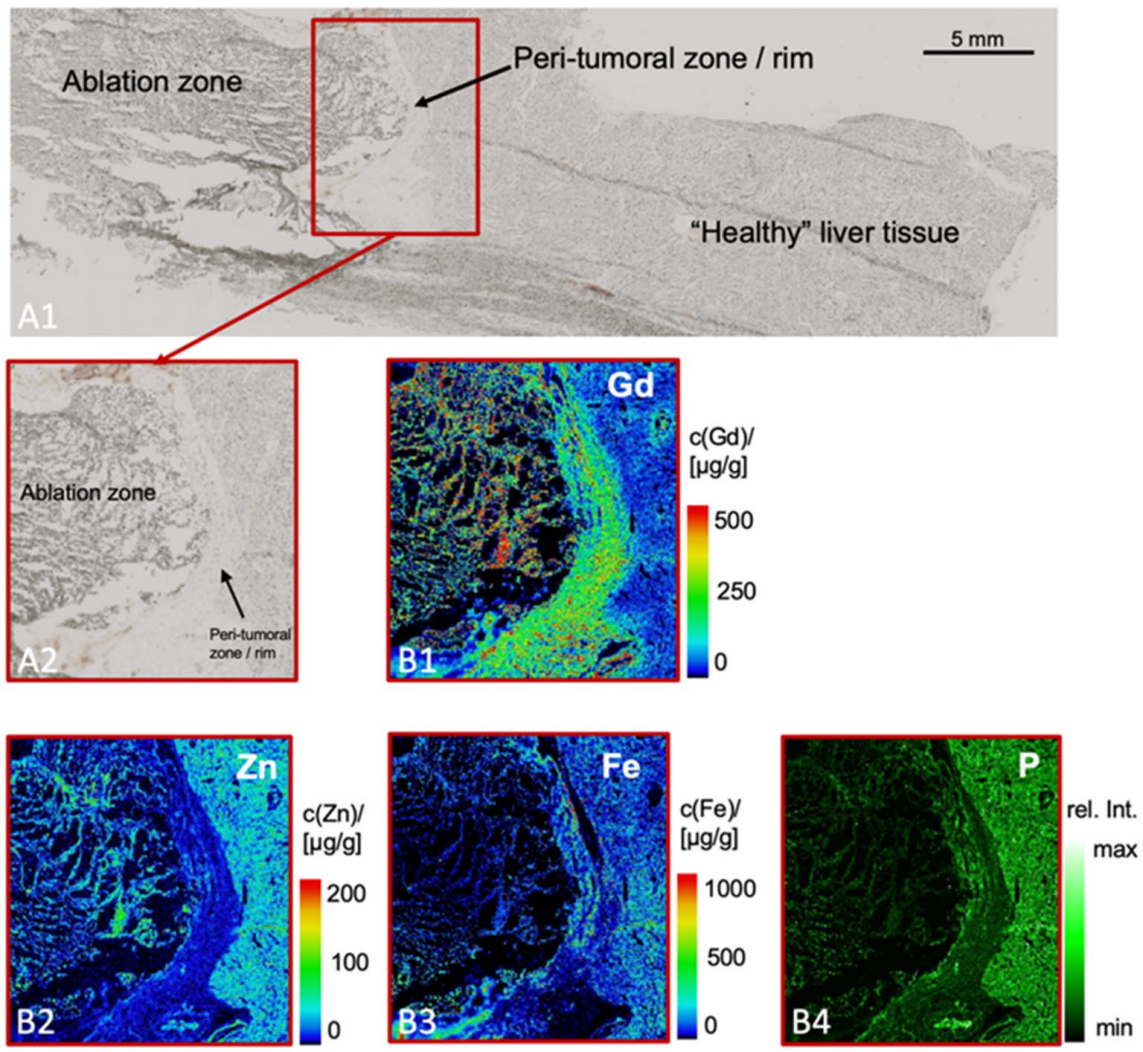
Laser ICP-MS for the assessment of the gadolinium distribution in the periablational rim. (**A1**,**A2**) Morphologic images show an overview of the location of the ablation zone, the periablational rim and the healthy liver tissue ((**A2**) magnification). (**B1**) Gadolinium-specific LA-ICP-MS confirmed the high signal of the elastin-specific probe in the periablational rim (green signal). (**B2**–**B4**) Zinc, iron and phosphorous measurements were performed as a control. These elements did not show a specific distribution pattern in the periablational rim. Scale bars represent 500 mm.

Laser-ICP-MS enabled the visualization of the element gadolinium on histological sections of the periablational rim. A clear co-localization of targeted gadolinium in the elastin-specific probe with elastic fibers could be confirmed, indicating a specific binding of elastic fibers (Fig. 3). The spatial distribution of potassium and sodium content in the samples were mapped as control, however, no specific distribution pattern was observed.

### Competition experiments

**Figure 4:**
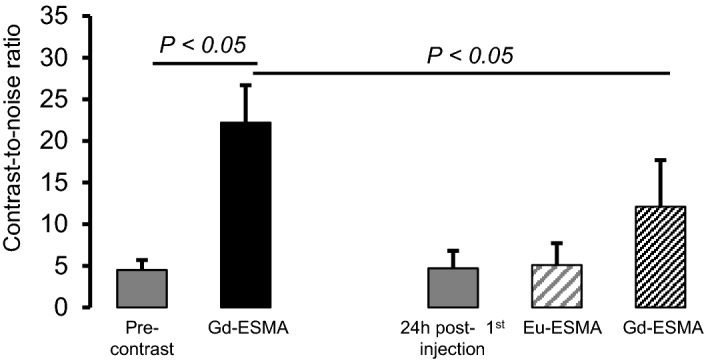
Competition experiments to demonstrate the specificity of the elastin-specific probe. The specific binding of the elastin-probe was confirmed by in vivo competition experiments in three animals three weeks after RF ablation. Following the injection of a tenfold higher dose of the non-paramagnetic europium-labeled elastin-specific-probe, a significant decrease of CNR was observed, compared to the administration of the gadolinium-labeled elastin-specific contrast agent alone indicating the specific binding of the probe.

To evaluate in vivo whether the elastin-specific probe binds specifically to elastic fibers in the periablational rim, a competition experiment was performed. The injection of a tenfold higher dose of a europium-labeled elastin-specific-probe resulted in no significant increase in CNR due to the non-paramagnetic characteristic of europium. Since the europium-labeled probe already blocked the binding sites of the elastic fibers, the gadolinium-labeled elastin-specific-agent could not bind properly, resulting in a significant decrease of CNR following the administration of ESMA on day two, compared to the administration of the gadolinium-labeled elastin-specific-agent alone on day one of the imaging protocol (Fig. 4), thus confirming a specific in vivo binding.

## Discussion

In recent years, substantial clinical and experimental data have shown that the effects of focal tumor ablation extend well beyond the ablation zone. While focal tumor ablation has been shown to expose in situ tumoral antigens and thus elicit specific immune response trough “in vivo vaccination” against the tumor^25–28^, ablation-induced secondary tissue reactions taking place in the rim surrounding the zone of ablation have been linked to the activation of pro-oncogenic pathways^6,16,17^. More specifically, the wound healing process implemented by the liver parenchyma in response to focal thermal ablation is characterized by an intense perifocal inflammation and a progressive deposition of structural proteins (in particular collagen and elastin) in the periablational rim resulting in a profound remodeling of the extracellular matrix with dramatic changes in the mechanical properties of the tumor microenvironment^6,16,29^. Along this lines, both in vitro and in vivo studies have shown that increased tissue stiffness significantly promotes the proliferation, motility and progression of heat-exposed residual HCC cells and that stiffness-dependent upregulation of the pro-proliferative ERK signaling cascade can be opposed by adjuvant therapies including Vitamin K1 and sorafenib^6,17–19,29–31^.

In light of these findings, a non-invasive imaging tool capable of visualizing and quantifying the processes of ECM remodeling in the periablational rim would have a dramatic impact in clinical practice allowing to identify patients at higher risk of tumor progression after RFA.

ECM remodeling and in particular elastin deposition has been previously imaged in vivo in the setting of cardiovascular disease using the gadolinium-based elastin-specific molecular probe ESMA^22,23^. Makowski et al*.* tested the use of ESMA-enhanced MRI for noninvasive assessment of plaque burden in a mouse model of atherosclerosis and showed that ESMA-enhanced MRI allows plaque characterization by quantifying intraplaque elastin content, establishing ESMA-enhanced MRI as a valuable tool for noninvasive assessment of plaque burden^22^. Recently, Keller et al*.* tested the use of the elastin-specific molecular agent ESMA to assess and quantify the peritumoral matrix in a rabbit VX2 hepatic tumor model. The authors demonstrated that ESMA-enhanced MRI enables the concise differentiation of the tumor center, margin and immediate peritumoral space with a higher accuracy compared to conventional gadolinium-based contrast agents in VX2 hepatic tumors (Keller et al. 2020).

The present study proves the feasibility of in vivo characterization of ECM remodeling following focal RFA using ESMA-enhanced MR imaging in a VX2 rabbit liver tumor model. The use of the elastin-specific probe ESMA allowed non-invasive quantification of elastin expression in the periablational rim and demonstrated a gradual increase of elastin deposits in the rim at different stages post-RFA. The quantifiable MR signal due to locally binding elastin-specific probe correlated with the histologically evaluated %EvG stain area, providing radiological-pathological ground truth validation for the imaging findings. In vivo CNR values after ESMA injection were in good agreement with ex vivo gadolinium concentrations as determined by ICP-MS indicating an actual increase of the elastin-specific probe in the rim throughout the different time points after ablation. Furthermore, the colocalization and specificity of the elastin-specific probe with elastic fibers were confirmed by Laser ICP-MS and competition experiments providing additional robustness to the data obtained from imaging and histopathology.

Our results pave the way for the use of ESMA-enhanced MRI to further study the post-ablation ECM in conjunction with other therapies causing changes of the tumor microenvironment. As such, investigating the role of elastin fibers as a potential scaffold for neo-angiogenesis or local tumor infiltration in a scenario of incomplete ablation would be of particular value for future research^31–33^.

Unlike existing clinical approaches, based on simple surrogates of tumor size or local enhancement to assess risks for tumor recurrence or response, ESMA-enhanced MRI may enable specific molecular-level assessment of the tumor microenvironment within the periablational zone. If translated into clinical scenarios, this imaging biomarker would be especially valuable when used in combination with other molecular imaging techniques e.g. capable of visualizing physiological conditions such as tumor pH or metabolic output^34,35^.

With regard to translation into clinical applications, the approach used in this study has different advantages. All imaging was performed on a clinical size 1.5 T MRI system. Therefore, the contrast agent’s relaxation, rotational correlation, and signal properties are readily available and can be immediately adapted to a clinical setting. The molecular structure and size of the probe are similar to contrast agents currently used in patients making adverse effects substantially less likely compared to larger molecules, e.g. antibodies and nanoparticles^22^. In turn, this may facilitate clinical approval. Furthermore, the clearance of the contrast agent is comparable to approved gadolinium-based MR contrast agents allowing for early imaging after injection with minimal background signal and thus maximal target to blood contrast to noise^22^.

Several limitations of our study need to be acknowledged. First, this study only includes a small number of rabbits. The number of animals per time point was prospectively determined by sample size calculation to avoid unnecessary waste of animal life. Second, we used a VX2 tumor model for HCC and not HCC tumor cells. However, this tumor model has several characteristics that make it similar to liver tumors and is hence frequently used for both ablation and imaging studies on liver tumors^36^.

In conclusion, ECM remodeling following tumor ablation can be readily delineated and quantified at different stages by elastin-specific molecular MR-imaging. Elastin-specific MRI could therefore provide important molecular information for a more accurate risk stratification of patient following RFA.

## Methods

### VX2 rabbit liver tumor model

Experimental procedures were conducted on New Zealand white rabbits (age: 11–17 weeks, weight: 3.0–3.5 kg, Charles River Laboratories, Sulzfeld, Germany) after approval of the regional Office for Health and Social Affairs of Berlin (LAGeSo) and in compliance with the regulations of the Federation of Laboratory Animal Science Associations (FELASA). The study was carried out in compliance with the ARRIVE guidelines. Animals were housed in a pathogen-free animal facility and kept in an environment with constant temperature and humidity under a 12-h phase light–dark cycle. Drinking water and food were available ad libitum. Twelve rabbits (experimental group) underwent implantation of VX2 tumor chunks in the left liver lobe as described in detail in previous studies^37–40^. Briefly, a freshly prepared suspension of cells derived from an established VX2 tumor cell line was injected in the muscle of the hind thigh of four donor rabbits (donor group). After confirmation of tumor growth by ultrasound imaging, donor animals were sacrificed, and the hind limb tumors were harvested. Macroscopically necrotic tissue was removed with a surgical blade and the tumor tissue was ground into 1 mm^3^ chunks. Tumor chunks were then implanted in the left liver lobe of the 12 experimental rabbits using an 18 G catheter during open laparotomy. The Glisson’s capsule was closed using an absorbable thrombogenic material (Gelfoam; Pfizer Inc., New York, USA) to circumvent peritoneal spread. The wound was then sutured in double layers. Tumor-bearing animals with a confirmed hepatic tumor growth between 1 and 2 cm at the implantation site were included in the study and underwent RFA of the hepatic VX2 tumor. All surgeries were performed under general anesthesia using s.c. medetomidine hydrochloride (Cepetor, 0.25 mg/kg; CP-Pharma, Burgdorf, Germany), and ketamine hydrochloride (Ketamin, 30 mg/kg, CP-Pharma, Burgdorf, Germany). Additionally, analgesic therapy was administered intravenously during surgery (buprenorphine [Temgesic, 0.03 mg/kg, Indivior Europe Limited, Dublin, Ireland] and subcutaneously (Carprofen [Rimadyl, 4.0 mg/kg, Zoetis, Berlin, Germany]) until 72 h after all surgical procedures.

### Experimental setup

**Figure 5:**
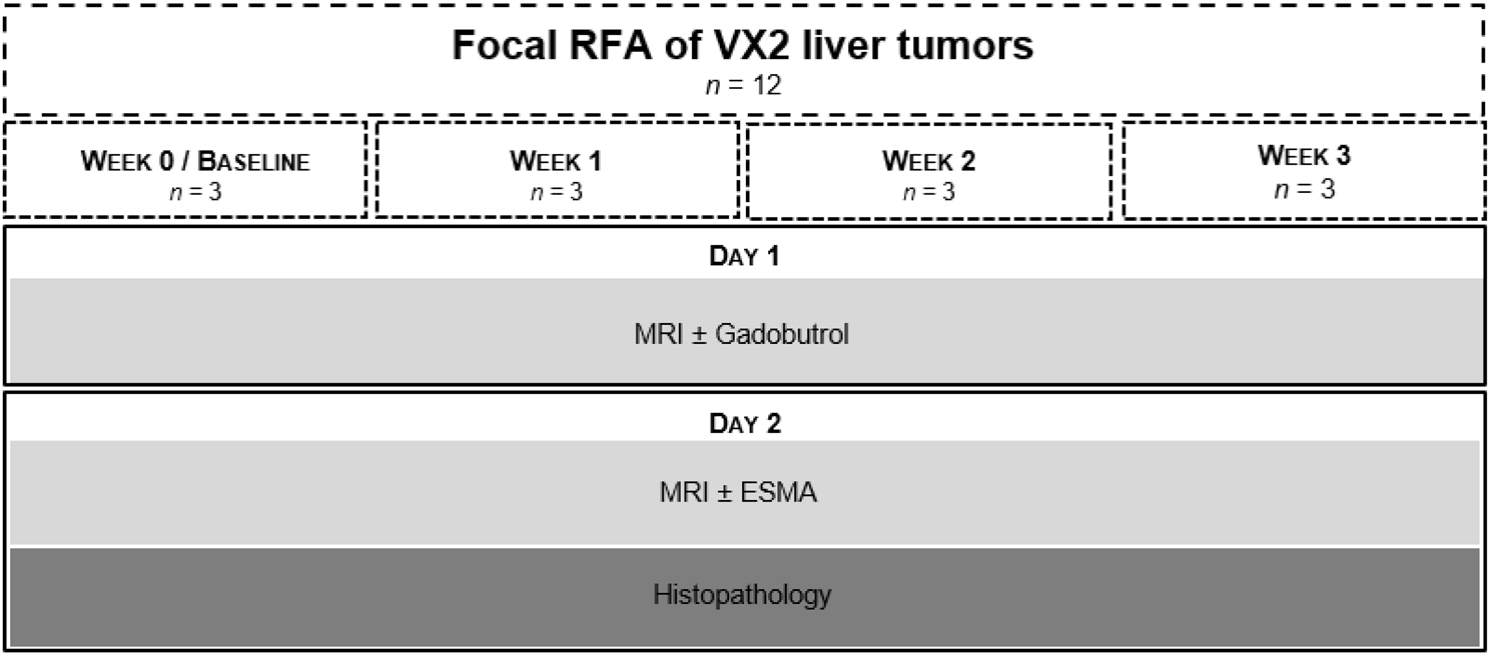
Experimental setup. The schematic diagram illustrates the MR imaging studies carried out on four experimental groups at consecutive time points. Imaging studies where completed: (I) at baseline on week 0 (n = 3; 1–2 days), (II) 1 week (n = 3; 7–8 days), (III) 2 weeks (n = 3; 14–15 days) and (IV) 3 weeks (n = 3; 21–22 days) after RF ablation. At each time point imaging studies were conducted on two consecutive days. On the first day, an MRI with and without the unspecific contrast agent Gadobutrol was performed. On day two of each time point, a second MRI scan with and without the elastin-specific probe ESMA was performed. After the ESMA-MRI animals were sacrificed and the livers were harvested for histologic analysis, ICP-MS and Laser ICP-MS.

The experimental setup is shown in Fig. 5. Following RF ablation animals were randomly assigned to four different groups and underwent MR imaging and subsequent necropsy and histopathologic evaluation at the following consecutive time points: (I) at baseline on week 0 (n = 3; 1–2 days), (II) 1 week (n = 3; 7–8 days), (III) 2 weeks (n = 3; 14–15 days) and (IV) 3 weeks (n = 3; 21–22 days) after RF ablation. Animals were scanned on two consecutive days for each time point. On the first day, MRI was performed before and following the injection of 0.2 mmol/kg gadobutrol (Gadovist, Bayer Healthcare AG, Berlin, Germany) which served as an unspecific control agent to test whether a significant unspecific binding occurs in the periablational zone. On the second day of each time point, the elastin-specific probe ESMA (Lantheus Medical Imaging, North Billerica, Massachusetts, USA) was administered at a dose of 0.2 mmol/kg. Following, MR imaging on day two of each time point animals were sacrificed using Pentobarbital-Natrium (Narcoren, Boehringer Ingelheim Vetmedica GmbH, Ingelheim, Germany), and their livers were harvested for histologic analysis.

### RF ablation

RFA procedures were carried out in a standardized fashion throughout the different time-points. Ablations were performed during laparotomy using a commercially available RF current generator (1500X RF, Angiodynamics, Latham, NY, USA). RF energy was delivered using a perfused 14-gauge RF needle applicator inserted centrally into the tumor to procure complete tumor ablation ^41^. Isotonic saline solution was continuously instilled into the coagulation zone via microbores in the needle tip at a flow rate of 40 ml/h. A self-adhesive neutral electrode was applied on the animal’s shaven back. To achieve standardization of the RFA procedures throughout the different time-points, the power output was calibrated to ensure a mean tip temperature of 70 °C for 5 min.

### MRI acquisition and specific binding of the ESMA probe

The elastin-specific molecular probe ESMA is a low molecular weight (856 g/mol molecular mass) gadolinium-based MR agent, used to image the ECM protein elastin^22,23^. The elastin-specific probe is composed of the d-amino acid d-phenylalanine, which is linked to a gadolinium-diethylenetriamine-pentaacetic-acid (Gd-DOTA) complex^22,42^. ESMA was used in several previous studies to visualize and quantify elastin fiber deposition in different organs^22–24^.

MRI was performed on a 1.5-T clinical MRI system (Avanto, Siemens Healthineers, Erlangen, Germany) and a clinically approved 4 channel knee coil. Rabbits were imaged in prone position under free-breathing conditions without cardiac or respiratory trigger gating system. MR scanning was performed before and 1 h after contrast agent injection. The imaging protocol included the following sequences. For an anatomical overview and localization of the liver, low-resolution three-dimensional gradient echo scout scan was performed. For the visualization and quantification of the molecular probe, a standard clinically used high-resolution T1 Turbo Spin-Echo sequence (TSE) was acquired before and after a bolus intravenous injection of the contrast agents of ESMA or Gadobutrol via the ear vein. The commercially available unspecific agent Gadobutrol served as control. Both ESMA and Gadobutrol were used in the same concentration of 0.2 mmol/kg body weight. Imaging parameters of the high-resolution T1 TSE sequence included a FOV = 160 × 160 mm, matrix = 240 × 240, in plane spatial resolution = 0.66 × 0.66, slice thickness = 2 mm, TR between subsequent IR pulses = 590 ms, and flip angle of 90°. In vivo competition experiments (n = 3) were performed to test the specificity of the binding of the elastin probe. MR imaging was performed within a 48 h imaging session. On the first day, the animals were imaged prior to and following the injection of the elastin-specific probe via the ear vein. After 24 h, imaging was performed prior to and following the injection of a tenfold higher dose of the non-paramagnetic europium-labeled elastin-specific MR probe. Subsequently, the elastin-specific probe was administered at a clinical dose of 0.2 mmol/kg and MR imaging was performed after 20 min.

### Image analysis

MR data analysis was performed by two radiologists (FC and MRM, both with more than 8 years of experience in advanced liver imaging) working in consensus. Morphometric measurements were implemented on high-resolution MR images using OsiriX (OsiriX Foundation, Geneva, Version 5.6, https://www.osirix-viewer.com/). To quantify MRI signal on ESMA-enhanced high-resolution T1 Turbo Spin-Echo sequence (TSE), three regions of interest (ROI) were placed in the rim of liver tissue surrounding the visible ablation zone (periablational rim) as well as in normal liver tissue on the contralateral lobe (normal liver tissue) at every time point. The mean value of the three regions of interest was then calculated. Background noise was determined as the standard deviation of air anterior to the liver. The CNR was calculated by the following formula: (SI _periablational rim_ − SI _normal remote liver tissue_)/Standard deviation noise.

### Histological analysis of ablation zones

Following surgical excision, the liver tissues were processed overnight in MorFFFix (Morphisto, Frankfurt am Main, Germany) and embedded in paraffin for sectioning. The section (5 µm) were stained for Miller’s Elastica van Gieson stain to visualize the elastic fibers. Hematoxylin and Eosin (H&E) staining were additionally performed. Resulting histological slices were examined using a light microscope (Observer Z1, Carl Zeiss Microscopy GmbH, Jena, Germany) and photographed. Computer-assisted image-analysis (ImageJ software, Version 1.51, NIH, https://imagej.nih.gov/ij/) of the digitized images of EvG sections was used for morphometry as well as for quantification.

### Laser ablation: inductively coupled plasma-mass spectrometry for elemental bioimaging

LA-ICP-MS was performed for quantitative elemental imaging of Gd, Fe, Zn, and P as described previously^43,44^. To determine the gadolinium distribution within the periablational rim following the injection of the elastin-specific gadolinium-based agent, x-ray spectra were acquired in the gadolinium distribution and the gadolinium distribution was mapped. Laser Ablation—Inductively Coupled Tissues were cut at − 20 °C into 10 µm cryosections and immediately mounted on SuperFrost Plus adhesion slides (Thermo Scientific). For LA-ICP-MS analysis, a LSX 213 G2 + laser system (CETAC Technologies, Omaha, USA) was used equipped with a two volume HelEx II cell and connected via Tygon tubing to an ICPMS-2030 (Shimadzu, Kyoto, Japan). To ablate the histological samples, a line-by-line scan with a spot size of 15 µm, a scan speed of 30 µm/s and 800 ml/min He as transport gas was performed. The analysis was performed in collision gas mode with Helium as collision gas and 100 ms integration time for the five analyzed isotopes 31P, 57Fe, 64Zn, 158Gd and 160Gd. For the quantification of Gd, matrix-matched standards based on gelatin were used. Nine gelatin standards (10% w/w) including a blank, were spiked with different Gd concentrations ranging from 1 to 5.000 µg/g. Averaged intensities of the scanned lines of the standards showed good linear correlation with a regression coefficient R^2^ = 0.9999 within this concentration range. Limit of detection (LOD) and limit of quantification (LOQ), calculated with the 3σ- and 10σ-criteria, were 8 ng/g and 28 ng/g Gd. The quantification and visualization were performed with an in-house developed software (WWU Münster, Münster, Germany).

### Mass spectroscopy

Inductively coupled mass spectroscopy (ICP–MS) was performed as previously described^45,46^ on a subset of liver samples (n = 3 per group). Samples were digested in 70% nitric acid at 37 °C overnight immediately after the last imaging session, followed by dilution with deionized water for ICP–MS analysis. A standard curve was acquired with each sample set for gadolinium concentration determination.

### Statistical analysis

The number of animals was determined by sample size calculation prior to the study. Statistical analysis was performed as previously described^43,45,46^. Values are expressed as mean ± standard deviation. The values of the different timepoints were compared with their controls using SigmaStat (Systat Software). A Student’s t test (unpaired, two-tailed) was applied for the comparison of continuous variables. In case of more than 2 groups, statistical comparisons were performed by analysis of variance (ANOVA) followed by the Bonferroni test. Univariate correlations were calculated using the Pearson correlation method. p < 0.05 was considered statistically significant.

## Data Availability

The datasets generated during and/or analyzed during the current study are available from the corresponding author on reasonable request.
